# Infection COVID-19 chez la femme enceinte: à propos d’un cas

**DOI:** 10.11604/pamj.2020.37.156.25233

**Published:** 2020-10-14

**Authors:** Sofiane Kouas, Olfa Zoukar, Khouloud Ikridih, Sameh Mahdhi, Ichrak Belghaieb, Anis Haddad

**Affiliations:** 1 Service de Gynécologie-Obstétrique, Centre Hospitalier Universitaire Tahar Sfar Mahdia, Mahdia, Tunisie,; 2 Centre de Maternité et de Néonatologie de Monastir, Monastir, Tunisie

**Keywords:** Infection COVID 19, femme enceinte, manifestations cliniques, prise en charge, COVID-19 infection, pregnant woman, clinical manifestations, care and support

## Abstract

La pandémie récente de COVID-19 a entrainé une crise sanitaire mondiale sans précédent. La susceptibilité des femmes enceintes aux infections a engendré beaucoup d´interrogations quant au sujet du risque de transmission, du risque tératogène et des complications materno-fœtales. La vitesse avec laquelle l´infection a progressé, ainsi que l´ambigüité de son impact sur la grossesse en raison de l´absence de données scientifiques a sollicité les obstétriciens à adapter leur pratique en se basant sur des conduites pragmatiques. Nous avons donné de l´intérêt à ce sujet déclaré par l´OMS comme pandémie mondiale en début mars 2020 pour essayer de planifier la prise en charge obstétricale des patientes infectées par SARS-CoV-2 sur la base des données actuelles. A partir d´un cas hospitalisé dans notre service de gynécologie Mahdia et se basant sur les connaissances actuelles; ce travail a pour objectifs de décrire les différentes manifestations cliniques possibles chez la femme enceinte et d´établir les principes de la prise en charge en cas d´infection COVID-19.

## Abstract (en)

The recent COVID-19 pandemic has led to an unprecedented global health crisis. The susceptibility of pregnant women to infections has raised many questions about the risk of transmission, teratogenic risk and maternal and fetal complications. The speed at which the infection advanced as well as the ambiguity effects on pregnancy, due to the absence of scientific data, led obstetricians to adapt their practice according to pragmatic approach. We focused on this subject declared by the WHO as a global pandemic in early March 2020 to develop a plan for obstetric management of SARS-CoV-2 infected patients on the basis of current data. Based on the literature and the case of a patient hospitalized in our Department of Gynecology in Mahdia, the purpose of this study was to describe the possible clinical manifestations of COVID-19 infection in pregnant women and to develop principles of management of patients with COVID-19 infection.

## Introduction

Les premiers cas d´infections dues à un nouveau coronavirus, le SARS-CoV-2 ont été enregistrés en Chine en décembre 2019. Cette maladie, désormais appelé COVID-19, a été déclarée comme pandémie par l´OMS trois mois après soit en mars 2020. A l´heure actuelle, plus d´un million de cas ont été objectivés à travers le monde occasionnant plus de 50 000 décès, en particulier en Europe.

Les modifications physiologiques immunologiques et cardio-pulmonaires liées à la grossesse font que les femmes enceintes soient plus vulnérables aux complications infectieuses et aux pathologies respiratoires. Des taux élevés de complications maternelles ont été recensés lors des précédentes épidémies de SARS-CoV et MERS-CoV. À l´heure actuelle, les données qu´on dispose concernant l´infection par SARS-CoV-2 sont rassurantes et n´indiquent pas de nombres d´infection plus élevés ni de risque surajouté de complications chez la femme enceinte par rapport à la population générale. Quelques exceptionnels cas de mortalité maternelle existent, mais surviennent le plus souvent sur des terrains qui présentent d´autres pathologies, particulièrement la prééclampsie. L´objectif de ce travail, réalisé à partir d´un cas hospitalisé dans notre service et en s´aidant des données récentes concernant cette infection COVID-19, est de souligner les manifestations cliniques possibles chez la femme enceinte tout en soulignant les principes de la prise en charge en cas d´infection COVID-19.

## Patient et observation

Il s´agit de Madame AB, âgée de 34 ans, G4P3A0 (3 enfants vivants dont un accouché par césarienne pour défaut d´engagement) consultant pour des douleurs pelviennes à type, des contractions utérines associées à des métrorragies noirâtres de faible abondance sur un terme de 29 SA + 3 jours. Par ailleurs, la patiente ne présentait pas d´antécédents familiaux ni médico-chirurgicaux particuliers. L´examen physique à l´admission était sans particularités notamment pas de contracture utérine ni des signes du choc hémorragique. L´examen au spéculum objectivait un saignement noirâtre minime d´origine endo-utérine. L´échographie pelvienne réalisée par voie sus-pubienne complétée par voie endovaginale avait montré un placenta praevia antérieur type 1 selon la classification de BESSIS avec un fœtus en position du siège eutrophique pour le terme. Par ailleurs; le liquide amniotique était de quantité normale et la longueur cervicale mesurée par voie endovaginale était de 30 mm.

Ainsi le diagnostic d´un placenta praevia associé à une menace légère d´accouchement prématuré était retenu et la patiente était hospitalisée et mise sous tocolyse per os avec une maturation pulmonaire fœtale. L´évolution était marquée par le tarissement du saignement et la disparition des douleurs pelviennes. Toutefois la patiente présentait à J3 d´hospitalisation une fièvre chiffrée à 41-42°C. L´examen clinique ne mettait pas en évidence un foyer infectieux et le bilan paraclinique infectieux réalisé de première intention est revenu négatif. Le diagnostic d´une infection COVID-19 a été évoqué surtout que l´interrogatoire approfondi révélait la notion d´un voisin infecté dans l´entourage lointain. La patiente était transférée dans l´unité d´isolement et un prélèvement par voie nasopharyngée était pratiqué et revenu positif le lendemain. La patiente était transférée par la suite au service COVID-19 et a reçu un traitement à base d´azithromycine avec une évolution favorable et un prélèvement de contrôle après une semaine revenu négatif. La patiente était mise sortante avec un RDV à notre consultation externe pour complément du suivi de grossesse.

## Discussion

La pandémie récente de COVID-19 a entrainé une crise sanitaire mondiale sans précédent. La vitesse avec laquelle l´infection a progressé, ainsi que l´ambigüité de son impact sur la grossesse en raison de l´absence de données scientifiques a sollicité les obstétriciens à adapter leur pratique en se basant sur des conduites pragmatiques. Les connaissances actuelles suggèrent qu´il n´y a pas de risque transmission materno-fœtale du COVID-19 [1]. Toutefois, il y a quelques cas publiés de nouveau-nés positifs qui sont en rapport avec des prélèvements effectués plusieurs heures après la naissance. Certaines études annoncent la possibilité d´une transmission verticale par la détection d´IgM dans le sérum des nouveau-nés issus des mères infectées, dont on sait que la spécificité est incertaine [2]. Cette annonce est controversée par l´absence de virus sur des prélèvements du liquide amniotique et du sang de cordon du nouveau-né. Ces données rassurantes concernent la majorité des cas d´infection qui surviennent au cours du 3^e^ trimestre de grossesse. Les informations relatives aux patientes exposées au début de la grossesse ne sont actuellement pas nombreuses [3].

Le risque que le virus soit tératogène semble peu probable. Toutefois, par analogie au SARS, l´infection à SARS-CoV-2 pourrait augmenter le risque de retards de croissance et de mort fœtale in utéro. En l´absence de données actuelles pragmatiques, la surveillance de la courbe de croissance fœtale notamment au cours du 3^e^ trimestre de grossesse est hautement recommandée [4]. Ceci est justifié sur la base des données d´une série de cas de 41 patientes atteintes de COVID-19 qui mettait en évidence un taux de 7% de pertes périnatales (2/41) et un pourcentage élevé d´accouchements prématurés de 41%. Toutefois, les indications à l´accouchement n´ont pas été clairement analysées au cours de cette étude [5] et toutes les données concernaient les patientes hospitalisées et donc a priori plus sévèrement atteintes. Cela suppose que cette étude n´avait pas inclus les patientes pauci ou pas symptomatiques ayant bénéficié d´une prise en charge extrahospitalière. Les manifestations cliniques (fièvre, toux, dyspnée, anosmie) sont similaires à celles chez les patientes non enceintes, bien que la fièvre semble moins présente que dans la population générale. Devant une telle symptomatologie notamment en cas de contact récent avec un sujet à risque dans la famille ou l´entourage, un test de dépistage par frottis nasopharyngé est justifié chez les patientes symptomatiques pour optimiser le suivi de la grossesse.

Sur le plan biologique, on observe fréquemment, une ascension des protéines de l´inflammation, une neutropénie concernant surtout les lymphocytes et une thrombopénie. Les explorations radiologiques notamment le scanner thoracique bien qu´elles ne soient pas obligatoires pour établir le diagnostic peuvent être d´apport pour orienter la prise en charge et ne doivent jamais être évités en raison de la grossesse car le pronostic vital maternel peut être mis en jeu dans les formes sévères. Ainsi et pour être pratique, une évaluation minutieuse de l´état clinique, la nécessité d´une hospitalisation voire même l´indication d´une admission dans une unité de soins intensifs (USI) peuvent être évalués à l´aide de scores cliniques dont les valeurs ont été adaptées à une population obstétricale [6]. Par ailleurs, beaucoup de sociétés savantes ont travaillé sur cette pandémie mondiale inquiétante et ont élaboré des guidelines et des recommandations sur la prise en charge obstétricale des patientes COVID-19 qu´il s´agit d´appliquer en fonction de la possibilité d´accueil et des conditions techniques de chaque unité de soins [7]. Les mesures de prévention primaire annoncées par l´OFSP contre cette infection et la transmission du virus, telles que l´éloignement social, l´hygiène rigoureuse des mains et le port obligatoire des masques pour la population générale, s´appliquent d´avantage aux femmes enceintes plus vulnérables.

La prise en charge des patientes malades de COVID-19 ne diffère pas du reste de la population en dehors de la pathologie obstétricale. Ainsi, les patientes pauci ou asymptomatiques peuvent être suivies en ambulatoire moyennant des contrôles téléphoniques dans un but d´assurer la continuité du suivi durant cette période. Pour les patientes symptomatiques et qui présentent une défaillance notamment respiratoire, une hospitalisation dans un service COVID-19 voire même en une unité des soins intensifs peut être justifiée.

Concernant les molécules utilisées, certaines équipes utilisent une corticothérapie dont l´impact sur l´évolution de la maladie maternelle n´est pas disponible. Cependant, toutes les sociétés savantes recommandent à l´unanimité de réaliser une maturation pulmonaire anténatale avant 34 semaines en cas d´accouchement inopiné avec un transfert in utéro vers un centre adapté en fonction de l´âge gestationnel, du poids fœtal estimé et des pathologies maternelles et ou fœtales détectées permettant une prise en charge du nouveau-né adapté à l´âge gestationnel. Par ailleurs, toutes les maturations pulmonaires prophylactiques devraient être abandonnées. La nécessité d´un traitement anticoagulant préventif est discutée, mais les données non publiées rapportent des taux élevés de complications thromboemboliques et ouvrent la question sur l´administration prophylactique systématique chez toute patiente enceinte hospitalisée avec une infection prouvée à SARS-CoV-2.

D´autres molécules (chloroquine, azithromycine, etc.) sont actuellement en cours d´essai clinique. Les bénéfices de ces traitements restent à prouver mais ces médicaments couramment utilisés au sein du Centre Hospitalier Universitaire (sous surveillance stricte notamment des effets indésirables) présentent un profil pharmacologique rassurant durant la grossesse, sans effets tératogènes connus [7, 8].

Du point de vue obstétrical, la voie d´accouchement ne devrait pas être influencé par la présence d´une infection à SARS-CoV-2, mais guidée par les indications obstétricales habituelles et l´état clinique de la patiente. Les efforts expulsifs peuvent être compromis par la gêne respiratoire et doivent être écourtés par l´utilisation d´un instrument (forceps, ventouse, spatule...). Le recours à une césarienne peut être indiqué chez les patientes qui présentent une détresse respiratoire (indication de sauvetage maternel). En cours de travail, une surveillance du rythme cardiaque fœtal et de l´état hémodynamique maternel doit être constante comme habituellement. Sur le plan analgésique, une anesthésie péridurale devrait être privilégiée pour diminuer le risque d´intubation lié à une anesthésie générale en cas de césarienne en urgence. La thrombopénie souvent observée en cas d´infection COVID-19 justifie un contrôle du taux des plaquettes de façon régulière notamment à l´entrée en salle de travail. Évidemment, des précautions particulières doivent être prises au sein du personnel soignant notamment minimiser le nombre de personnes en contact avec une patiente infectée et insister sur le port de matériel de protection tel que blouses et masques. Dans notre établissement, nous préconisons des mesures supplémentaires par port de masques ultra-filtrants (FFP2) et de lunettes lors de l´accouchement, en raison du risque d´aérosolisation.

L´allaitement présente d´importants effets bénéfiques pour le développement du nouveau-né et pour le lien mère-enfant. À ce jour, aucun cas de transmission lié à cette pratique n´a été décrit et le virus n´a pas été retrouvé dans le lait maternel. Ainsi, une infection COVID-19 ne représente pas une contre-indication à l´allaitement si les précautions lors du soin au nouveau-né, telles que le lavage des mains, la désinfection du sein et le port du masque sont respectées.

## Conclusion

Le monde entier en particulier médical s´est retrouvé face à une nouvelle situation inquiétante lors de cette pandémie COVID-19. Les recommandations et les directives sur la prise en charge des patientes enceintes infectées COVID-19 obéissent à de perpétuelles modifications et ceci essentiellement en raison des données scientifiques récentes et des essais cliniques en cours. L´enregistrement de différentes données dans un registre international permettra dans l´avenir de mieux cerner les risques obstétricaux liés au COVID-19 et d´éclaircir les principes thérapeutiques les plus efficaces pour faire face à cette pandémie.
